# Similar range of motion and function after resurfacing large–head or standard total hip arthroplasty: 2–year results from a randomized clinical trial

**DOI:** 10.3109/17453674.2013.788435

**Published:** 2013-05-31

**Authors:** Jeannette Østergaard Penny, Ole Ovesen, Jens–Erik Varmarken, Søren Overgaard

**Affiliations:** ^1^Department of Orthopaedic Surgery and Traumatology, Odense University Hospital, Odense; ^2^Department of Orthopedic Surgery, Naestved Hospital, Naestved; ^3^Institute of Clinical Research, University of Southern Denmark, Odense, Denmark.

## Abstract

**Background and purpose:**

Large–size hip articulations may improve range of motion (ROM) and function compared to a 28–mm THA, and the low risk of dislocation allows the patients more activity postoperatively. On the other hand, the greater extent of surgery for resurfacing hip arthroplasty (RHA) could impair rehabilitation. We investigated the effect of head size and surgical procedure on postoperative rehabilitation in a randomized clinical trial (RCT).

**Methods:**

We followed randomized groups of RHAs, large–head THAs and standard THAs at 2 months, 6 months, 1 and 2 years postoperatively, recording clinical rehabilitation parameters.

**Results:**

Large articulations increased the mean total range of motion by 13° during the first 6 postoperative months. The increase was not statistically significant and was transient. The 2–year total ROM (SD) for RHA, standard THA, and large–head THA was 221° (35), 232° (36), and 225° (30) respectively, but the differences were not statistically significant. The 3 groups were similar regarding Harris hip score, UCLA activity score, step rate, and sick leave.

**Interpretation:**

Head size had no influence on range of motion. The lack of restriction allowed for large articulations did not improve the clinical and patient–perceived outcomes. The more extensive surgical procedure of RHA did not impair the rehabilitation.

This project is registered at ClinicalTrials.gov under # NCT01113762.

The traditional total hip arthroplasty (THA) with a polyethylene/metal articulation requires postoperative restrictions due to the risk of dislocation and recommendations of a moderate level of activity. Younger patients have higher activity, which leads to increased polyethylene wear (Schmalzried et al. 2000)—a factor that contributes to poor implant survival in this age group, with a revision frequency of approximately 15–20% after 15 years (Karrholm et al. 2008, Overgaard et al. 2008). Metal–on–metal (MoM) articulations can limit the volumetric wear (Anissian et al. 1999) and have the advantage of a large head size either as large–head THA or resurfacing hip arthroplasty (RHA). A large head may give better function by increasing the range of motion (ROM) (Bader et al. 2004, Burroughs et al. 2005, Davis et al. 2007), particularly for the large–head THA as apposed to RHA (Bengs et al. 2008, Kluess et al. 2008).

The reduced risk of dislocations (Beaule et al. 2002, Burroughs et al. 2005, Padgett et al. 2006) allows patients with large articulations unrestricted movement postoperatively. It causes the patient less anxiety (Nissen et al. 2011) and permits more intensive training, which has led to faster recovery when using the anterolateral approach (Kuijer et al. 2009, Sharma et al. 2009). An RHA requires a larger incision than THA (Vendittoli et al. 2006, Swank and Alkire 2009). Mini incisions rather than standard ones have shown a few immediate advantages, but no lasting effect on function (Sharma et al. 2009). However, surgical detachment of the distal part of gluteus maximus to access the acetabulum could affect muscular function and increase pain and blood loss—which could delay early and permanent function.

A fast and full recovery is important for the well–being of the patient; in addition, there is a strong association between the length of sick leave and the likelihood of expulsion from the work force (The Danish ministry of Occupation 2001). Since most younger patients are employed, a speedy recovery is important for return to work.

**Figure:**
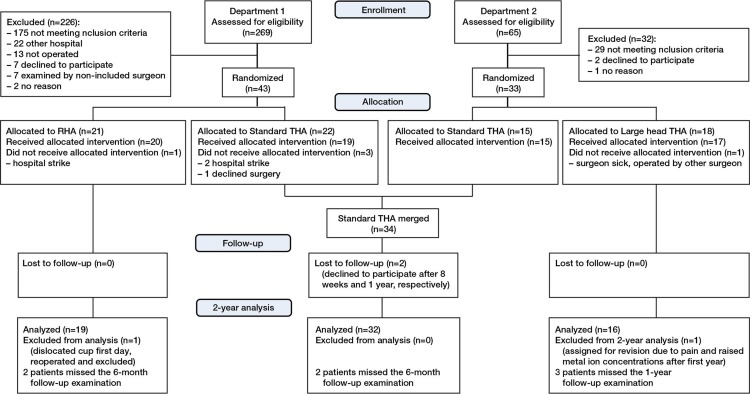
Consort flow chart of the inclusion and follow–up. There is a discrepancy between the number of assessments in each department, as one of the departments screened the GP referrals prior to assignment to the project. If the referral mentioned one of the exclusion criteria, they were never assessed.

In this randomized clinical trial (RCT), the primary endpoint was range of motion for 2 years following surgery due to osteoarthritis, comparing RHA, large–head THA, or standard THA. Our secondary endpoints were clinical function (measured using Harris hip score (HHS), UCLA activity, WOMAC, EQ–5d, and step rate during the first 2 years) and rate of return to work.

## Patients and methods

### Sample size and inclusion criteria

The sample size was based on range of motion as measured with a goniometer. This method is not exact (Holm et al. 2000), which is why we considered that a difference of 45 degrees represented a true difference. Assuming a type–I error of 5% and a type–II error of 20%, and a minimal relevant difference, the sample size was calculated to be 16 patients in each of the 3 groups.

The trial was performed in compliance with the Helsinki Declaration and was approved by our ethics committee (project–ID: VF–20050133, Nov. 9, 2005; the Ethical Review Board, Funen County, Denmark). Following receipt of oral and written information, all patients gave their informed consent.

The patients were enrolled at 2 departments. One (Hospital South, Naestved, Region Zealand, Denmark) used randomization to standard THA or large–head THA and the other (Odense University Hospital, Denmark) used randomization to standard THA or RHA. All the authors participated in finding the patients and then JOP included them in the study.

Sealed envelopes (prepared by a nurse) were used for the allocation. Odense had 2 surgeons and the envelopes were block–randomized with half of each intervention for each (10 + 10), and in Naestved one surgeon operated all the patients. Upon inclusion, the patient picked out an envelope and the intervention was known to the patient and staff before surgery, which was necessary due to the need for different instruments for each patient group.

Both departments had identical inclusion and exclusion criteria. The inclusion criteria were primary osteoarthritis, secondary osteoarthritis due to mild dysplasia, and age from 40 to 65 years. Exclusion criteria were: dysplasia with CE angle of < 25 degrees on the AP projection, severe femoral head deformation, reduced femoral neck length, leg length discrepancy of more than 1 cm, need for restoration of offset, deformation after fractures or earlier osteotomies, a previous hip arthroplasty, inflammatory arthritis, endocrine disease with metabolic manifestations in bone, renal disease, malignant disease, neuromuscular or vascular disease of the affected leg, osteoporosis (BMD < 2.5 SD), use of opioid pain killers due to other diseases, high–dose corticosteroids, BMI of > 35, pregnant or planning to be, or problems that could prevent completion of our follow–up program. (Figure). The patients were operated between April 2007 and December 2009.

### Prosthetic components

The RHA group received an Articular Surface Replacement (ASR; DePuy, Leeds, UK) made from a high–carbon cobalt–chromium–molybdenum alloy. The median head size for resurfacing was 51 (47–57) mm. The study only included head sizes from 47 mm and over due to the increased risk of revision with smaller heads reported at the time the study began (Amstutz et al. 2004). The large–head THA group received an M2aMagnum/ReCap articulation made from cast high–carbon cobalt–chromium (median head size: 50 (44–56) mm) in combination with a cementless forged titanium Bimetric stem.

The standard THAs at Odense University Hospital received a titanium Mallory Head acetabular shell with an Arcom Ringlock polyethylene liner, and a 28–mm Biolox delta modular ceramic head, mounted on a titanium Bimetric stem (all Biomet, Bridge End, UK). At Hospital South, Naestved, the standard THA group received a 28–mm cobalt–chromium head and a titanium Trilogy CH cup, in combination with a VerSys Fiber titanium metal taper stem. The first 9 patients got a Trilogy UHMWPE liner and the last 6 got a Longevity liner (Zimmer, Warsaw, IN).

### Surgery

Spinal analgesia was used in most patients. All patients were administered identical antibiotic, bleeding, and antithrombotic prophylaxis.

The RHA was implanted according to the manufacturer’s instructions through a posterolateral approach. The gluteus maximus muscle was divided, and the insertion was detached along with the external rotators. The cup was placed press–fit without cement, 1 mm under–reamed, and the femoral component was cemented with SmartSet™ GHV bone cement (DePuy). The other cups were inserted reamed size–to–size and the femoral stem was inserted using standard instruments, all through an identical posterolateral approach but with smaller incision and without gluteal muscle release. We aimed for a cup inclination of 45° with 20° anteversion. Tantalum markers were inserted around the acetabulum and the proximal femur for radiostereometry, causing increased surgery time for RHA and large–head THA.

### Rehabilitation

Postoperative rehabilitation included full weight bearing in all patients. Apart from allowing free range of motion in the RHA and large–head THA groups, the hospital physiotherapists gave identical instructions to all patients in a home–based training program. If the patients asked for supervised training at the 8–week check–up, we referred them to a council–based training facility (with a similar hip program to that they had already followed, supervised). Some patients may have been referred through their GP. Patients were informed that they could expect an average sick leave of 2–3 months but were told that they could resume work earlier if possible.

### Outcome instruments

During hospitalization, we recorded the surgery time (incision to close, and time used for tantalum marking), blood loss, length of incision, intraoperative complications, preoperative and postoperative (second–day) hemoglobin levels, and length of stay.

Clinical function was evaluated in the out patient clinic preoperatively and at 8 weeks, 6 months, 1 year, and 2 years. ROM was evaluated by a single trainee orthopedic surgeon (JOP), with a view to controlling pelvic tilt. A clear 18–cm plastic goniometer was used. Extension was tested in the prone position and flexion, abduction, adduction, and internal and external rotation were tested in the supine position, the latter two with the hip and knee flexed 90°. Function was scored with the Harris hip score (HHS) and the UCLA activity score (Zahiri et al. 1998), ranging from 1–10 where 10 is best.

As activity levels can be difficult to assess using a questionnaire (Zahiri et al. 1998), we supplemented this with walking activity measured with the Yamax (YX200) pedometer (Schneider et al. 2003) 1 week before each follow–up visit. Self–reported quality of life was rated using the EQ–5D (Wittrup–Jensen et al. 2009) with values from –1 to +1, where +1 is the optimum health state, and the self–reported function was rated with the WOMAC questionnaire (Bellamy et al. 1988) using a VAS scale, with values from 0 to 100 where 0 is the best result.

Patients available for the work force were asked their occupations and the date for returning to their job. The job classifications placed them in 7 socioeconomic groups (Spieker and Plougsing 1997), but due to the low numbers, we analyzed “desk jobs” (SOCIO 11 + 131–133) and “manual labor” (SOCIO 134, 135, and 2) based on the physical demands of the job.

### Statistics

As a result of randomizing at 2 departments, we had 2 standard THA groups. Despite having different bearing materials, they were merged to 1 standard THA group as we regarded them as being equal with respect to rehabilitation.

Demographic data are presented as median (range). They were compared by the Kruskal–Wallis test. Clinical data are presented as mean (SD). The intraoperative data and longitudinal baseline data were analyzed by ANOVA (t–test between 3 groups) and follow–up data with ANCOVA adjusted for the baseline values. To facilitate comparison between the 3 groups, the mean difference (95% CI) between standard THA and RHA, between large–head THA and RHA, and finally between large–head THA and standard THA were determined. Fisher’s exact test was used to evaluate differences between the distributions of job classes, and a stratified log–rank test was used to compare the length of sick leave. All baseline/ANOVA tests were adjusted for center effect/geographic location. STATA 11.2 software (StataCorp LP, College Station, TX) was used for all analyses and p–values less than 0.05 were considered statistically significant. A biostatistician supervised the data handling.

## Results

The groups were comparable, but the large–head THA group was somewhat older than the other patients, and the proportion of females was less in the THA group (Table 1). Blood loss and decrease in hemoglobin were similar in the 3 groups (Table 2). The RHA patients were hospitalized 3.6 (1.8) days on average, the large–head THA patients 4.2 (1.5) days, and the standard THA patients 3.7 (1.2) days (p = 0.8).

**Table 1.** Demographic data. Age and BMD are presented as median (range)

	RHA	Standard THA	Large–head THA
n	20	34	17
Age, years	57 (54–61)	56 (52–62)	63 (54–64)
Females, %	40	29	47
Charnley class I, %	80	69	59
BMI, kg/m**^2^**	28 (24–31)	27 (25–29)	28 (26–32)

**Table 2.** Surgery–related outcomes, presented as mean (SD). Surgery time for RHA and large–head THA patients included a mean 12–min (a) and 8–min (b) surgery time due to insertion of tantalum markers related to a radiostereometry study. Tantalum time was missing for two–thirds of the RHA patients, so adjusted surgery time is not reported. Subtraction of mean tantalum times for missing values also finds a group difference in adjusted surgery time with p–values < 0.001

	RHA	Standard THA	Large–head THA	p–value
n	20	34	17	
Surgery time, min	113 (15)**^ a^**	66 (11)	83 (12)**^ b^**	< 0.001
Incision length, cm	24 (2.8)	14 (2.8)	15 ( 2.6)	< 0.001
Blood loss, mL	625 (467)	515 (216)	753 (315)	0.09
Hemoglobin decrease, mmol/L	1.7 (0.7)	1.8 (0.5)	2.2 (0.6)	0.5

Regarding complications, the RHA group had one cup displacement with dislocation on the day after surgery. The patient had the cup repositioned, and he was excluded from further follow–up. 1 RHA patient was prescribed peroral antibiotics by the GP, who suspected infection around the skin clips. The investigators were not involved in the treatment of the patient, and infection was not confirmed. A large–head THA patient suffered a pulmonary embolus following a deep vein thrombosis. She recovered, but with a slightly reduced lung capacity. Another large–head THA patient experienced pain and raised levels of metal ions after the first year. The patient was reoperated, and subsequently excluded. The THA group experienced 3 dislocations, all of them occurring within the first 2 postoperative weeks. All 3 patients were treated with closed reduction. None of them have had recurrences, and none were excluded.

The ROM over time only differed statistically significantly in a few directions at irregular points in time (Table 3). The primary endpoint was total ROM, and after 2 and 6 months the large articulations improved the total ROM by up to 13 degrees compared to standard THA—but the difference was not statistically significant (p = 0.5 for RHA and 0.6 for large–head THA). During the following 18 months, the standard THA value continued to improve slightly, the large–head THA value remained the same, and the RHA value declined slightly, resulting in all 2–year total ROM values being within 9 degrees of each other (p = 0.6). The adduction movements had improved 6 and 4 degrees more for RHA and large–head THA than for the standard THA (p = 0.02) after 8 weeks, and with RHA the difference increased to 7 degrees at 6 months (p = 0.03) whereafter the difference disappeared. The standard THA had an extra 4 degrees of extension at 8 weeks compared to large–head THA (p = 0.07). The large–head THA extension caught up with that in the other groups after 8 weeks, but the gap reappeared at 2 years, with 3 degrees less extension than standard THA and 5 degrees less than RHA (p < 0.01). The RHA abduction increased less at 8 weeks than in the other groups (p = 0.4), but then it seemed to overtake by a few degrees at 6 months (p = 0.8). At 2 years, however, the abduction had declined for RHA but had increased further for the other groups, leaving a statistically significant difference of 4 and 6 degrees to standard and large–head THA, respectively (p = 0.02). The flexion, internal rotation, and external rotation results were not statistically significantly different during follow–up.

**Table 3.** Hip movements compared by regression analysis adjusted for baseline values. The mean difference (95% CI) measured in degrees between standard THA and RHA, large–head THA and RHA, and large–head THA and standard THA was calculated

	A	B	C	D	E	F			
*Baseline*									
ROM, total	156 (31)	161 (44)	145 (29)	5	(–16 to 25)	–11	(–31 to 8)	–16	(–37 to 5)
Extension	–1 (3)	1 (4)	2 (4)	2	(–0 to 4)	2	( –0 to 4)	0	( –2 to 3)
Flexion	90 (12)	91 (13)	85 (13)	1	(–6 to 8)	–5	(–13 to 3)	–6	(–14 to 1)
Abduction	26 (12)	25 (11)	22 (8)	–1	(–8 to 5)	–5	(–11 to 2.0)	–3	( –9 to 2)
Adduction	14 (7)	14 (9)	13 (6)	–0	(–4 to 4)	–1	( –5 to 4)	–1	( –5 to 4)
Internal rotation	5 (9)	6 (14)	2 (10)	2	(–5 to 8)	–3	( –10 to 3)	–5	(–12 to 2)
External rotation	22 (8)	24 (12)	22 (9)	2	(–3 to 7)	0	( –5 to 6)	–2	( –8 to 4)
*2 years*									
ROM, total	221 (35)	232 (36)	225 (30)	8	(–11 to 28)	9	(–12 to 31)	1	(–17 to 19)
Extension	7 (8)	5 (6)	3 (4)	–2	(–6 to 1)	–5	(–9 to –2) **^a^**	–3	(–6 to –0) **^a^**
Flexion	103 (12)	107 (14)	103 (10)	3	(–4 to 10)	2	(–6 to 9)	–1	(–8 to 6)
Abduction	31 (5)	36 (10)	36 (8)	4	(0 to 9) **^a^**	6	(1 to 10) **^a^**	2	(–3 to 6)
Adduction	24 (6)	24 (7)	24 (5)	0	(–4 to 4)	–0	(–4 to 4)	0	(–4 to 4)
Internal rotation°	25 (10)	25 (9)	24 (10)	–1	(–6 to 4)	1	(–5 to 8)	2	(–3 to 8)
External rotation°	30 (9)	35 (8)	35 (9)	5	(–0 to 9)	4	(–2 to 11)	0	(–6 to 5)

A RHA: Mean (SD) B Standard THA: Mean (SD) C Large-head THA: Mean (SD) D Standard THA minus RHA: Mean difference (95% CI) E Large-head THA minus RHA: Mean difference (95% CI) F Large-head THA minus standard THA: Mean difference (95% CI) ^a^ CIs not including zero

For all groups, the secondary outcomes improved between baseline and 6 months postoperatively (Table 4), but after that progress was limited. The difference in HHS was within 5 points at all times, and at 6 months the mean HHS for THA was above 90. At 1 year, all means had reached the “excellent” category. The 28–mm THA group had a relatively large baseline step rate and reached steady state at 6 months, whereas the large articulations improved in step rate up to 2 years and reached a similar level. Higher UCLA scores of 7.4 were observed for the RHA patients within the first 6 months, whereas the large–head THAs (with 6.9) lagged behind the other groups for the first 6 months before overtaking standard THA. Patients with large articulations reported more strenuous activity of half a UCLA point after 1 year, and 1 point after 2 years, compared to standard THA, but the differences were not statistically significant (p = 0.7 and 0.2, respectively).

**Table 4.** Clinical endpoints compared by regression analysis adjusted for baseline values. The mean difference (95% CI) between standard THA and RHA, large–head THA and RHA, and large–head THA and standard THA was calculated

	A	B	C	D	E	F			
*Baseline*									
HHS	63 (10)	56 (9)	59 (8)	–7	(–13 to –2) **^b^**	–4	(–10 to 2)	3	( –2 to 8)
WOMAC **^a^**	50 (21)	55 (16)	49 (20)	6	(–6 to 17)	–1	(–15 to 13)	–6	(–18 to 5)
UCLA activity	5.8 (2.2)	6.3 (1.8)	5.8 (1.6)	0.5	(–0.7 to 1.7)	0.0	(–1.2 to 1.3)	–0.5	(–1.5 to 0.5)
Steps, mill/y	1.8 (0.9)	2.3 (1.5)	1.6 (0.6)	0.5	(–0.2 to 1.2)	–0.2	(–0.7 to 0.4)	–0.7	(–1.3 to –0.0)
Eq–5d	0.6 (0.3)	0.6 (0.2)	0.6 (0.2)	0.1	(–0.1 to 0.2)	0.0	(–0.1 to 0.2)	–0.0	(–0.1 to 0.1)
*2 years*									
HHS	93 (10)	91 (14)	95 (6)	2	(–3 to 8)	4	( –1 to 9)	2	(–3 to 7)
WOMAC **^a^**	8 (13)	10 (18)	5 (6)	1	(–8 to 10)	–4	(–11 to 3)	–5	(–11 to 2)
UCLA activity	7.3 (1.8)	7.0 (2.0)	7.8 (1.7)	–0.6	(–1.6 to 0.4)	0.4	(–0.8 to 1.6)	1.0	(–0.1 to 2.1)
Steps, mill/y	3.0 (2.0)	2.8 (1.4)	2.5 (1.2)	–0.1	(–0.8 to 0.5)	0.2	(–0.6 to 0.9)	0.3	(–0.6 to 1.2)
Eq–5d	0.8 (0.3)	0.9 (0.2)	1.0 (0.1)	0.0	(–0.1 to 0.2)	0.1	(–0.0 to 0.2)	0.1	(0.0 to 0.2) **^b^**

A–F: See Table 3 **^a^** WOMAC can be reported from 0 to 100 and a low score represents minimal discomfort. A negative mean difference in the columns comparing large–head THA and standard THA or RHA indicates that large–head has less discomfort. For all other endpoints, a high score is optimal. **^b^** CIs not including zero

The patient–reported outcomes also improved drastically after surgery. The WOMAC scores reported less than half of the original discomfort as early as 3 weeks after surgery, and only around one–fifth after 8 weeks. At 6 months, patients with small articulations reported more discomfort than large–head THA patients (7 points of difference) and RHA patients (3 points of difference) (p < 0.01) After that, the improvement slowed down and there was little change in patient–perceived function over the following 18 months. The patients with large articulations still reported slightly less discomfort, but the difference was not significant (p = 0.3). There was no significant difference between groups regarding quality of life up until 2 years, where the patients with large–head THAs (minus the excluded patient) scored slightly better (p < 0.05).

The mean (SD) sick leave period for RHA, standard THA, and large–head THA was 87 (54), 101 (80), and 141 (92) days, respectively. Despite the fact that there was seemingly a large difference favoring the RHA patients, we could not validate this statistically in this small population (p = 0.8).

## Discussion

In this RCT, the outcome after insertion of a resurfacing, large–head, or standard total hip arthroplasty in patients with osteoarthrosis showed no clinically relevant difference with regard to ROM, HHS, WOMAC, UCLA activity, EQ–5D, step rate, or sick leave.

The strength of our study was the randomized design, with standardized follow–up and clearly defined inclusion and exclusion criteria. The major limitation was the small numbers. We could not demonstrate the sought–after 45–degree difference in ROM. More patients and the use of a more reliable measurement method will be needed in order to achieve smaller differences. The primary endpoint was ROM. With more secondary endpoints comes the risk of false statistical significance, and our few statistically significant findings must be interpreted with caution. An example may be the significant differences in some subgroups of the total ROM, both at baseline and at further follow–ups. Despite the statististical significance, the differences were small and of doubtful clinical importance.

The unblinded design is another drawback that was chosen from fear of dislocation, of which we saw 3 in the standard THA group. This is a clear limitation of the study, not only for UCLA activity and ROM but also for WOMAC, where the fear of dislocation may have influenced the standard THA group negatively. The development of stronger polyethylene—allowing the use of larger standard heads—should minimize the risk of dislocation, and could allow for blinding in future studies.

One other limitation of the study was the gluteal muscle detachment that was performed on all RHA patients to standardize the study. Experienced surgeons can avoid it in many cases; thus, our data reflect an RHA group that has undergone more extensive surgery than the average RHA group. We were unable to demonstrate any negative effect of the larger muscle injury from RHA, but we cannot be sure whether muscle–sparing surgery would have improved the RHA outcomes.

Range of motion was the primary outcome, and we could not demonstrate any advantage of the unrestricted regime allowed for the large articulations. During the first 6 months, we observed a small difference favoring the large articulations, but the larger head size may not have been the cause. The difference could partly be explained by improvement in adduction and improvement in internal rotation, i.e. the movements the standard THA group was prohibited from during the first 8 weeks. The pattern with very similar 2–year changes in ROM indicates that the restraints could have limited the initial flexibility of the soft tissue, so once the restrictions were lifted and time had passed, the patients became less aware of the hip and used it more naturally—allowing the standard THA group to catch up with the larger articulations.

Randomized studies are scarce, but our RHA results regarding ROM match those from other prospective studies (Hing et al. 2007, Dela Rosa et al. 2007, Hakkinen et al. 2010). Larger 2–3 year retrospective comparisons between RHA and standard THA have favored both THA (Stulberg et al. 2010) and RHA (Vail et al. 2006), but only by a few degrees, which was unlikely to have been of clinical importance. Randomized studies between THA and large–head THA (Zijlstra et al. 2011), or between RHA and standard THA (Howie et al. 2005), generally support our results—with only a few degrees separating the groups. Neither study found a difference in total ROM between the groups, and the overall 1–year ROMs were similar to what we found.

A study with comparison between 3 prospective groups of THA, large–head THA, and RHA reported a better ROM in the large–head THA group, where the advantage was seen in flexion and when rotation was measured in the prone position (Lavigne et al. 2011). We found supine rotations similar to their maximum rotations (prone), but whether our large–head THA patients would have shown even larger rotation if tested in the prone position remains a question. The study by Lavigne et al. lacked randomization, but was strengthened by larger numbers, single (investigator) blinding, and by using a more reproducible method to measure ROM. The finding of increased flexion for large–head THA is interesting, as it is consistent with a theoretically better ROM for a large head–to–neck ratio, but it is not supported by our results, which are more supportive of the suggestion that in time, all hips return to a predestined ROM (Le Duff et al. 2009).

Despite the fact that most studies have been unable to demonstrate differences in ROM between interventions, the improvement in ROM in itself is related to a better patient–evaluated outcome (Bryant et al. 1993, Davis et al. 2007), and Lavigne (Lavigne et al. 2011) found both flexion and total ROM to correlate to low WOMAC scores. Spearman analyses were not part of our original protocol, but when performed we found constant negative correlations ranging from –0.040 to –0.378 (statistically significant at 8 weeks and 2 years) at all follow–up times (data not shown). Not being part of the protocol, it can only be used to generate hypotheses, but the implication of a better function with increased ROM should prompt more research, not only in developing favorable implants, but also to look into physiotherapy to improve ROM. Surgery improves but does not restore ROM to healthy hips (Hakkinen et al. 2010, Lavigne et al. 2011), and with better rehabilitation programs patients might experience greater or faster improvement.

Despite the larger incision/surgical procedure for RHA, our secondary outcome measures generally failed to demonstrate a clinical disadvantage. A non–significant delay in reaching excellent HHS was noted, and as the surgical detachment of the gluteal muscle delays recovery of maximal lower limb muscle strength (Jensen et al. 2011), it may have affected the limp or distance score. The observed difference, however, was only 5 points and had disappeared by 1 year; and the larger incision did not prolong the hospitalization or impair the early ROM, WOMAC or UCLA. Apart from an overall (unexplained) lower step rate in all groups, our results support other short–term studies of RHA and standard and large–head THA (Vail et al. 2006, Vendittoli et al. 2006, Bohannon 2007, Daniel et al. 2009, Stulberg et al. 2010, Zhou et al. 2009, Garbuz et al. 2010, Lavigne et al. 2010, Garellick et al. 2011, van der Weegen et al. 2011, Costa et al. 2012) where little clinical difference was observed.

Our patients had longer mean sick leaves than those reported in the UK (Mobasheri et al. 2006), but they were slightly older and included some on sick leave before surgery. In contrast to a larger randomized study (Vendittoli et al. 2006), we failed to demonstrate a faster return to work for the large articulations, and contradicting our hypothesis of a quicker rehabilitation for restriction free implants, the large–head THA patients had the longest sick leave despite having the lowest WOMAC scores. One possible explanation for this conflicting result would be that the large–head THA group were a little older and more likely to have a manual job than other groups, so despite reporting low disability, the demands of the job may have exceeded the WOMAC tasks and caused them to postpone their return to work.

The ASR RHA used in our study has been withdrawn from the market due to above–average failure rates, but apart from the initial cup dislocation, the RHA patients are all functioning well with clinical outcome measures similar to other brands of RHA, so it seems reasonable to draw conclusions on the RHA as a concept rather than a specific type.

Overall, we found similar outcome for RHA, standard THA, and large–head THA during the first postoperative years, and we were unable to demonstrate any clear benefit from lack of postoperative restrictions. The very similar end–stage functional level just above UCLA 7 in both this and other RHA studies could indicate that the average patient just reaches his/her desired level of activity at 6 months and has no interest in participating in more strenuous activities than bicycling—despite having an implant that is supposed to cope with more strain. From the present study, we cannot advocate the use of RHA or large–head MoM THA with regard to clinical outcome. Careful patient selection when choosing RHA or large–head THA candidates is called for, as more short–term complications are associated with this type of implant (Johanson et al. 2010, Graves et al. 2011, Porter et al. 2012). Whether large–head THA using different bearing materials may be advantageous to the patients is beyond the scope of this paper.
